# Minimizing comprehensive geriatric assessment to identify deterioration of physical performance in a healthy community-dwelling older cohort: longitudinal data of the AEQUIPA Versa study

**DOI:** 10.1007/s40520-020-01562-8

**Published:** 2020-05-01

**Authors:** R. Diekmann, S. Hellmers, L. Elgert, S. Fudickar, A. Heinks, S. Lau, J. M. Bauer, T. Zieschang, A. Hein

**Affiliations:** 1 Assistive Systems and Medical Device Technology, Department of Health Services Research, Carl von Ossietzky University of Oldenburg, Oldenburg, Germany; 2 Peter L. Reichertz Institute for Medical Informatics of TU Braunschweig and Hannover Medical School, Hannover, Germany; 3 Geriatric Medicine, Heidelberg University, Agaplesion Bethanien Krankenhaus Heidelberg, Ruprecht-Karls-Universität Heidelberg, Heidelberg, Germany; 4 Department of Geriatric Medicine, Klinikum Oldenburg, Carl von Ossietzky University of Oldenburg, Oldenburg, Germany

**Keywords:** Primary prevention, Frailty, Comprehensive geriatric assessment, Older adults, Muscle power test, Mobility tests

## Abstract

**Background:**

It is important to identify the relevant parameters of physical performance to prevent early functional decline and to prolong independent living. The aim of this study is to describe the development of physical performance in a healthy community-dwelling older cohort aged 70+ years using comprehensive assessment over two years and to subsequently identify the most relevant predictive tests for physical decline to minimize assessment.

**Methods:**

Physical performance was measured by comprehensive geriatric assessment. Predictors for the individual decline of physical performance by Principal Component and *k*-means Cluster Analysis were developed, and sensitivity and specificity determined accordingly.

**Results:**

*2*51 subjects (Ø 75.4 years) participated in the study. Handgrip strength was low in 21.1%. The follow-up results of tests were divergent. Handgrip strength [− 16.95 (SD 11.55)] and the stair climb power test (power) [− 9.15 (SD 16.84)] yielded the highest percentage changes. Four most relevant tests (handgrip strength, stair climb power time, timed up & go and 4-m gait speed) were identified. A predictor based on baseline data was determined (sensitivity 82%, specificity 96%) to identify subjects characterized by a high degree of physical decline within two years.

**Discussion:**

Although the cohort of older adults is heterogeneous, most of the individuals in the study exhibited high levels of physical performance; only a few subjects suffered a relevant decline within the 2-year follow-up. Four most relevant tests were identified to predict relevant decline of physical function.

**Conclusion:**

In spite of ceiling effects of the geriatric assessment in high-performers, we assume that it is possible to predict an individual’s risk of physical decline within 2 years with four tests of a comprehensive geriatric assessment.

## Background

Physical function is of major relevance for older people’s quality of life, cognitive state and independence in activities of daily living (ADLs) [1–3]. Decline in physical function leads to disability in older adults [4], physical activity is associated with greater physical function [5]. As the percentage of older people in our communities steadily grows, it is becoming increasingly important to understand physical function in a bid to avoid care dependency and disability. Loss of independence and the inability to perform ADLs impose a growing burden on the health care system. In general, “physical performance”, including parameters of function, strength/power, balance and endurance, in older people is usually reviewed by comprehensive geriatric assessments such as handgrip strength, short physical performance battery, timed up & go, and stair climb power test. Choosing the most sensitive and meaningful instrument for assessing physical performance is a major challenge, particularly because the group of subjects 70+ years is very heterogeneous.

Cut-off values and clinically meaningful changes for the risk of functional decline and adverse health outcomes have already been defined for several of the aforementioned established tests. For walking speed, for instance, a cut-off of 0.8 m/s was identified as being associated with adverse health outcomes [6]. Taking more than 20 s for the timed up & go test is associated with low mobility [7]. Participants who took longer than 16.7 s to rise from a chair five times represented the slowest quartile in a cohort of 1122 subjects and exhibited the highest percentages of four-year follow-up disability [8]. Muscle strength and muscle power are important determinants of physical performance and mobility skills in older adults [9–11]. While strength is defined as the ability to exert force, muscle power is defined as the ability to exert force over time (power = force × velocity) [9]. Both parameters have significant effects on the fear of falling and the quality of life [12], and play a special role in the screening and diagnosis of sarcopenia [13], a disease that may lead to a loss of independence. Even a single measurement of handgrip strength has shown to be predictive of health outcomes [14]. Using data from the Women’s Health and Aging study, Xue et al. sought to predict the risk of falling, physical disability, and frailty by the rate of decline in grip strength. However, they concluded that greater baseline handgrip strength was significantly associated with a lower risk of IADL disability and frailty [15]. McKinnon et al. postulated that muscle power declines earlier than muscle strength, due to a reduction of motor unit numbers with aging [16]. Therefore, muscle power may exert a greater influence on physical performance than strength [9, 10]. Leg muscle power can be estimated from chair rising or stair climbing [17, 18]. Common tests to assess leg muscle power in older adults are the five times chair rise test (which is part of the short physical performance battery) [4] and the stair climb power test [18]. The five times chair-rise test has proven useful in clinical decision-making, although it exhibits limitations to discriminate good and poor performers in terms of balance disorders [19]. Many older adults are unable to perform the stair climb power test due to orthopedic or neurologic problems, a lack of power or fear of falling [20]. Many tests in the geriatric context exhibit ceiling effects. Therefore, a distinction in high-performers is challenging.

To our knowledge, only few previous studies have addressed longitudinal data on the physical performance of a high-performing population 70+ , measured by a comparably broad battery of assessments, including function, strength and power, balance and endurance with emphasis on the individual course of physical decline. In view of high functional level of the study population we did not focus on established geriatric thresholds, but rather on changes over time since we believe it is of utmost importance in the sense of primary prevention to detect an individual’s risk at a very early stage. Additionally, most studies have investigated younger subjects or subjects with a wider age range [21]; conducted fewer physical tests; monitored the follow-up examination only by (telephone) interview; or did not consider individual trajectories.

### Objective

The present longitudinal observation Versa study (prediction for maintaining self-employment in old age) of older independent community-dwelling people aged above 70 years was part of the primary prevention project called AEQUIPA (physical activity and health equity: primary prevention for healthy aging). The aim of the study was to describe the development of physical performance in a high-performing group over two years, measured by comprehensive geriatric assessment, and to identify the most predictive tests for individual deterioration to minimize this assessment. This may be useful for identifying individuals with the highest risk who may benefit from early intervention, e.g. fitness programs.

## Methods

### Study population

Community-dwelling older adults without any acute health problems participated in the study. Recruitment took place in sports clubs, senior appointments, music societies, rehab sport centers, physiotherapy departments and via newspaper advertisements. The study inclusion criteria were: a minimum age of 70 years; community-dwelling; no severe acute diseases (e.g. lung, kidney or heart); no difficulties in climbing a flight of ten steps; the ability to attend assessments independently; no pacemaker or other electronic implants; and a timed up & go test < 20 s.

### Study design

In this longitudinal observational study, eligible participants were assessed at the study center at the Carl von Ossietzky University Oldenburg for baseline assessment (*t*_0_). A written informed consent sheet was sent to the participants after a phone call at least one week before the baseline evaluation. All subjects signed informed consent. Subsequent visits were made after six (*t*_1_) and 24 (*t*_2_) months; the tests outlined below were performed in a standard manner each time. A health history was recorded including a semi-structured questionnaire for health status (hypertension, past strokes, chronic diseases as e.g. diabetes mellitus and COPD, falls and general health) and medication review. Blood pressure was measured for safety reasons, a value of > 180/95 mm/Hg led to a termination of the study of the affected participant. The study protocol was approved by the Medical Ethics Committee of the Hannover Medical School (MHH) (Nr. 6948), Germany.

### Physical performance

*Handgrip strength (HGS)*: HGS was measured using a JAMAR hand-held dynamometer (Jamar, Bolingbrook, IL). The participants performed the test while seated, using both hands and alternating with three trials per hand. The maximum of the mean value of the three measurements of the left or right hand (whichever was the stronger) was taken. Reduced HGS according to frailty criteria was related to body mass index (BMI) and defined according to Fried et al. [22].

*The stair climb power test (SCPT):* SCPT was used to measure leg power. The time taken (in sec.) (SCPTT) for the subject to climb a flight of ten stairs was measured, and the related power was calculated (in watts, P = m × g × h/t) (SCPTP) according to Bean et al. [18].

*The timed up & go test (TUG)*: The TUG is an established test in community-dwelling older people to reliably assess functional mobility and its clinical change over time [23]. TUG was measured in seconds, and the results were evaluated according to Podsiadlo and Richardson [7].

*Short physical performance battery (SPPB):* SPPB included the five-times chair rise test *(5TCR)*, 4-m gait speed *(4mGS)* and balance tests (semi-tandem stand, tandem stand) according to Guralnik et al. [8]. The time (in seconds) taken to perform each component and the cumulative score were used to assess the results.

*The six minute walking test (6mWT)*: 6mWT is a common test for assessing functional exercise capacity and endurance performance over a period of time [24]. In this study, the participants were instructed to walk continuously at their individual habitual pace along a 20 m corridor until the tester asked them to stop; distances were recorded in meters (m). For safety reasons, the instructor accompanied the subject for the full distance. The time taken was measured in seconds using a stopwatch.

### Statistical analysis

To describe the cohort, data were given in absolute numbers and in percentages as the mean, standard deviation, minimum, maximum, and the 1st to 3rd quartile, respectively. The results of follow-up data were presented in percentages, with a negative sign for a deterioration of physical performance and a positive, respectively no sign for an improvement. Friedman test, a non-parametric statistical test, was used to detect differences across multiple test attempts. A *p* value of ≤ 0.01 is referred to be statistically significant.

#### Reduction of the number of assessments

A principal component analysis (PCA), also known as orthogonal transformation, is a statistical procedure in the exploratory statistic and multivariate data analysis with the aim to convert a set of observations of possibly correlated variables into a set of linearly uncorrelated variables called principal components. The idea of this analysis is to “reduce the dimensionality of a data set, which consists of a large number of interrelated variables, while retaining as much as possible of the variation present in the data set” [25]. In the present study, PCA was applied to identify the most relevant components—and therefore of assessments—of physical performance and to reduce the number of variables of the comprehensive geriatric assessment. Non-metric measures as the categorical variable “semi- and tandem stand” were excluded from PCA [26].

#### Sub-group identification

The data was visualized using vector graphs. A *k*-means cluster analysis [27] was used on the basis of *t*_2_ data to divide n observations into k clusters in which each observation belongs to the cluster with the nearest mean. In the present analysis, clustering was used to identify subjects with comparable characteristics regarding physical function to find subjects with different levels of function according to the reduced set of assessment. The appropriate number of clusters was decided based on content, as described later.

#### Individual predictive value

Three hypothetical predictors have been developed to identify subjects with low function (see “Results”: Cluster 4 contains subjects with the lowest functional status). For Predictor 1 we used the cluster centers of the area of lowest physical function and adjacent clusters at *t*_2_ to identify the position and orientation of the dividing lines. We then plotted the position of the subjects at *t*_0_ and identified which subjects were already in the low function area (Cluster 4) at the baseline. Mean values of vectors of each subject of each cluster have been calculated. For Predictor 2, according to these mean values of vectors of the area of lowest physical function and adjacent clusters at *t*_0_ new dividing lines have been calculated. Then the individual position of the subjects at the baseline *t*_0_ was plotted and they matched to the cluster of lowest physical function. For Predictor 3, examining the delta values of *t*_0_–*t*_1_, subjects were considered if they deteriorated in terms of strength (*y*-axis) and mobility (*x*-axis); in the following, the procedure was identical to that of Predictor 2. Sensitivity and specificity for subjects of lowest physical function (“test positive”) were calculated for all three predictors.

#### Software

Statistical analysis was performed using IBM Corp. Released 2017, IBM Statistics for Windows, version 25.0, Armonk, NY: IBM Corp. and Matlab R2019 (The MathWorks Inc.).

## Results

We included 251 participants (mean age 75.4 years) in the study at baseline; 148 (59%) women and 103 (41%) men. High blood pressure was present in 51.8%, diabetes in 8.4%, past stroke and COPD in 6.8%. Table 1 shows the baseline and follow-up characteristics. Four (1.6%) subjects dropped out of the study after six months, and a further 19 (in total 23 = 9.1%) after 24 months. The majority of the subjects (*n* = 196, 78.1%) had an age-associated normal BMI between 20 and 30 kg/m^2^; one (0.4%) subject was malnourished (BMI < 20 kg/m^2^); and *n* = 54 (21.5%) were obese (BMI > 30 kg/m^2^). According to the established geriatric threshold, *n* = 53 (21.1%) of the subjects exhibited a low handgrip strength at the baseline, *n* = 67 (26.7%) reached less than 3 points in the 5TCR test and none of the subjects needed more than 20 s for the timed up & go test (according to inclusion criteria) (data not shown).

**Table 1:** Characteristics at the baseline (*t*_0_), after six months (*t*_1_) and after 24 months (*t*_2_)

	*n* (%)	Min	Max	Mean (SD)	1st to 3rd quartile	*p* value Friedman test
Age (years)						
* t*_0_	251	70	87	75. 1 (3.89)	72.00–77.00	
* t*_1_	247	70	88	75.87 (3.84)	73.00–78.00	
* t*_2_	226	71	89	77.23 (3.78)	74.00–79.00	
Female						
* t*_0_	148 (59)					
* t*_1_	146 (59)					
* t*_2_	132 (58)					
BMI (kg/m^2^)						
* t*_0_	251	19.20	42.20	27.43 (4.10)	24.50–29.40	0.912
* t*_1_	247	19.50	42.40	27.40 (4.11)	24.50–29.40	
* t*_2_	226	19.50	40.70	27.23 (3.96)	24.40–29.23	
Physical function						
HGS (kg)						
* t*_0_	251	10.67	55.33	28.87 (10.04)	21.33–37.00	0.000
* t*_1_	247	10.00	53.67	28.54 (9.79)	21.33–36.00	
* t*_2_	225	7.00	48.67	24.32 (9.57)	17.00–31.00	
Female (kg)						
* t*_0_	148	10.67	35.00	22.13 (4.76)	18.41–25.59	
* t*_1_	145	10.00	34.33	22.17 (5.10)	18.33–26.00	
* t*_2_	132	7.00	30.00	17.91 (4.43)	14.75–21.00	
Male [kg]						
* t*_0_	103	22.00	55.33	38.56 (7.31)	34.50–42.67	
* t*_1_	102	18.33	53.67	37.59 (7.43)	33.00–43.08	
* t*_2_	93	14.00	48.67	33.42 (7.23)	29.17–38.50	
SCPTT (s)						
* t*_0_	251	3.33	10.64	5.85 (1.17)	5.08–6.39	0.000
* t*_1_	247	3.28	12.27	5.83 (1.14)	5.09–6.38	
* t*_2_	220	3.20	14.29	6.21 (1.25)	5.48–6.81	
SCPTP (W)						
* t*_0_	251	101.24	456.67	218.66 (50.71)	185.20–247.93	0.000
* t*_1_	246	102.04	401.82	218.74 (48.94)	188.49–245.06	
* t*_2_	220	78.67	368.50	202.48 (47.50)	172.28–229.37	
TUG (s)						
* t*_0_	251	4.95	14.05	8.50 (1.68)	7.40–9.38	0.000
* t*_1_	247	5.04	15.26	8.49 (1.73)	7.22–9.15	
* t*_2_	225	5.34	16.63	8.95 (1.85)	7.67–9.94	
4mGS (s)						
* t*_0_	251	1.61	4.38	2.78 (0.49)	2.47–3.07	0.001
* t*_1_	247	1.59	4.74	2.73 (0.52)	2.38–2.98	
* t*_2_	224	1.76	5.90	2.80 (0.54)	2.45–3.05	
5TCR (s)						
* t*_0_	251	6.70	23.89	12.44 (3.04)	10.40–13.75	0.001
* t*_1_	246	6.32	21.38	12.00 (2.82)	9.94–13.66	
* t*_2_	220	6.44	20.26	11.70 (2.66)	9.81–13.24	
SPPB (pts.)						
* t*_0_	251	7	12	10.97 (1.04)	10–12	0.359
* t*_1_	247	8	12	10.96 (1.16)	10–12	
* t*_2_	224	3	14	10.83 (1.39)	10–12	
SPPB semi-tandem (pts.)						
* t*_0_	251	0	1	0.99 (0.09)	1–1	0.013
* t*_1_	247	1	1	1.00 (0.00)	1–1	
* t*_2_	224	0	1	0.97 (0.17)	1–1	
SPPB tandem (pts.)						
* t*_0_	251	0	2	1.91 (0.31)	2–2	0.000
* t*_1_	247	0	2	1.81 (0.44)	2–2	
* t*_2_	224	0	2	1.64 (0.64)	1–2	
6mWT (m)						
* t*_0_	251	198.00	640.00	435.50 (74.14)	391.00–480.00	0.000
* t*_1_	247	245.00	655.00	442.11 (74.87)	398.00–485.00	
* t*_2_	209	70.00	610.00	429.84 (79.35)	392.50–470.50	

*BMI* body mass index, *HGS* handgrip strength, *SCPTT* stair climb power test (time), *SCPTP* (Power), *TUG* timed up & go, *4mGS* 4 m gait speed, *5TCR* 5 times chair rise, *SPPB* short physical performance battery, *6mWT* 6 min walk test, *SD* standard deviation

Table 2a, b show percentage changes in physical performance tests compared to the baseline for the first and second follow-up, respectively, with the highest percentage changes at the beginning. The highest percentage changes in the first follow-up occurred in 5TCR [2.18 (SD 17.41)%] and 6mWT [1.70 (SD 8.18)%], and in the second follow-up in HGS [− 16.95 (SD 11.55)%] and SCPTT [− 9.15 (SD 16.84)%]. The changes present a decline of function when sign is negative and an improvement when sign no sign is present, which means a positive sign.

**Table 2:** Changes of physical performance (percentage)

		*n*	Min	Max	Mean (SD)	1st to 3rd quartile
	% Delta of:					
	A. *t*_0_–*t*_1_ in descending order					
1	5TCR	246	− 60.72	47.82	2.18 (17.41)	− 8.19–13.18
2	6mWT	247	− 28.37	34.48	1.70 (8.18)	− 3.22–6.37
3	4mGS	247	− 50.43	38.07	1.02 (12.22)	− 5.45–8.21
4	SCPTT	247	− 92.92	32.90	− 0.97 (13.60)	− 7.36–7.85
5	TUG	247	− 70.89	30.78	− 0.74 (13.54)	− 8.12–7.89
7	HGS	247	− 39.70	36.10	− 0.64 (11.76)	− 8.43–5.79
8	SCPTP	246	− 48.99	52.28	0.54 (12.48)	− 6.75–8.02
9	SPPB	247	− 27.27	28.57	0.19 (9.11)	− 8.33–9.09
	B. *t*_0_–*t*_2_ in descending order					
1	HGS	225	− 55.27	18.52	− 16.95 (11.55)	− 23.49 to − 9.23
2	SCPTT	220	− 104.27	35.60	− 9.15 (16.84)	− 16.76–1.89
3	SCPTP	220	− 46.08	56.59	− 7.17 (13.73)	− 14.68–1.30
4	TUG	225	− 53.48	26.83	− 6.71 (14.82)	− 15.57–4.03
5	5TCR	220	− 72.13	41.49	2.78 (18.21)	− 9.26–16.47
6	4mGS	224	− 88.50	26.57	− 2.48 (14.24)	− 8.61–5.96
7	6mWT	209	− 82.05	36.21	− 2.25 (12.72)	− 8.36–4.36
8	SPPB	224	− 70.00	22.22	− 1.53 (11.29)	− 8.33–9.09

A negative sign (−) means a deterioration and no sign an improvement

*HGS* handgrip strength, *SCPTT* stair climb power test (time), *SCPTP* (Power), *TUG* timed up & go, *4mGS* 4 m gait speed, *5TCR* 5 times chair rise, *SPPB* short physical performance battery, *6mWT* 6 min walk test, *SD* standard deviation

According to the Friedman test, data differs significantly across the three measurements (significance level of *p* < 0.01, data not shown), except for BMI, SPPB and the SPPB tandem stand test. Only subjects with completed data at all three time points were included in the analysis (*n* = 208).

Table 3 shows the results of PCA (t_2_ data) to identify the most relevant variables (assessments) for describing physical performance. BMI, SPPB and the SPPB semi-tandem stand test were excluded from further analysis because they did not differ over time or they were ordinally scaled.

**Table 3:** Principal component analysis (PCA) at (*t*_2_)

	Component 1	Component 2
SCPTT	0.912	0.031
TUG	0.905	0.230
4mGS	0.887	0.119
6MWT	− 0.800	− 0.029
5TCR	0.660	0.494
SCPTP	− 0.675	0.569
HGS	− 0.368	0.836

*HGS* handgrip strength, *SCPTT* stair climb power test (time), *SCPTP* (Power), *TUG* timed up & go, *4mGS* 4 m gait speed, *5TCR* 5 times chair rise, *6mWT* 6 min walk test

Two main components were identified regarding the 24-month follow-up: first (*x*-axis), a combined time axis strongly associated with mobility measured via the variables SCPTT, TUG and 4mGS; second (*y*-axis), a component dominated by HGS. A cut-off value of > 0.8 was set for the integration of variables.

**Fig. 1:**
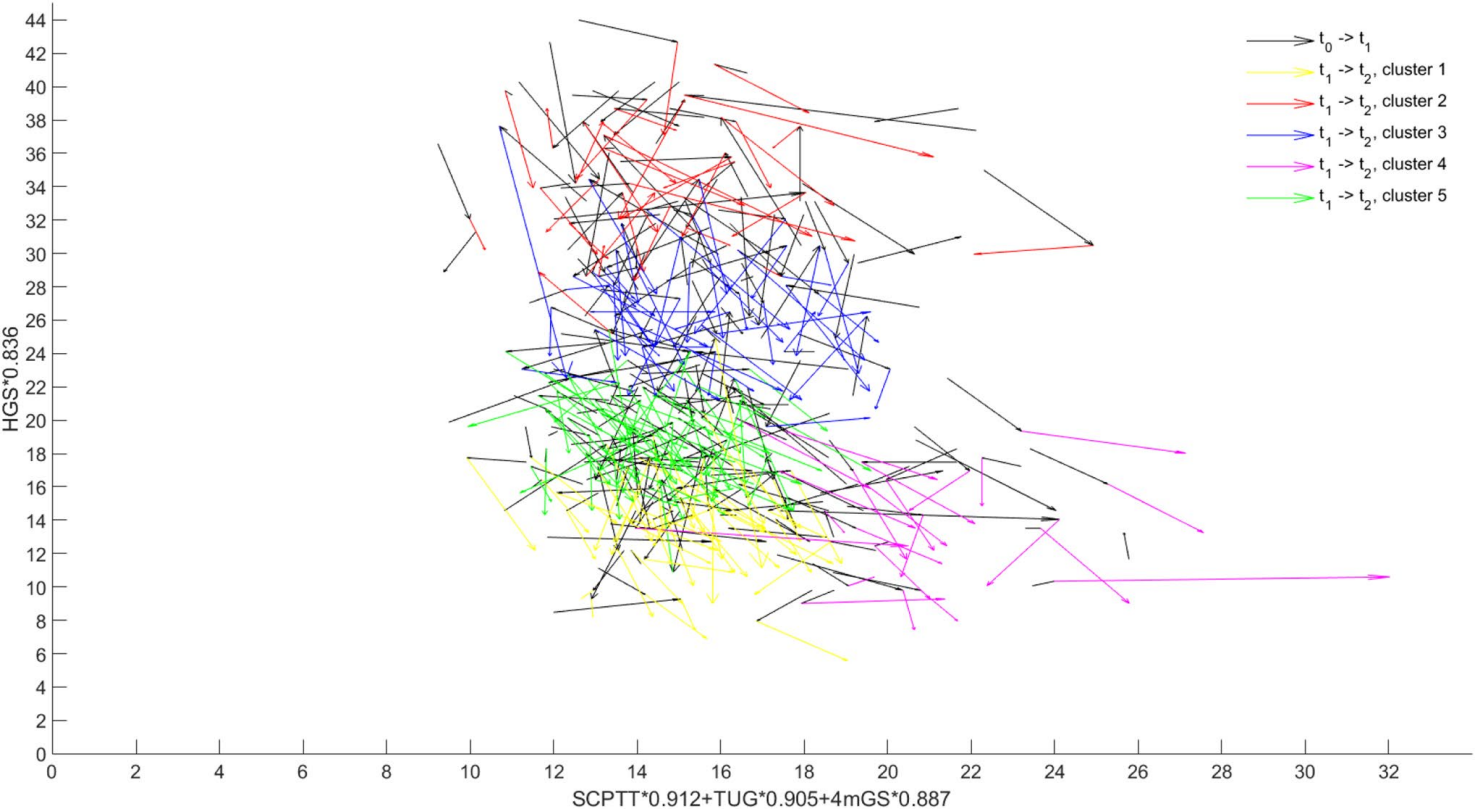
Vector graph showing physical function at all three time points (black line *t*_0_–*t*_1_, *t*_1_–*t*_2_ colored according to the five clusters)

The data of vector position at *t*_2_ was analysed by *k*-means cluster analysis to identify sub-groups of comparable physical function. We decided to continue analysis with five clusters for issue-based reasons and due to a lack of a qualitative criterion. Three clusters included only men or only women. In four clusters, delta values (*t*_0–_*t*_1_) of physical performance differed only weakly. Six clusters resulted in an uneven distribution in terms of the number of subjects in each cluster. The silhouette coefficient, the only established quality criterion for cluster analysis, was approximately comparable in all cluster number variations (approx. 0.5 on a scale between − 1 and + 1) (data not shown). Figure 1 presents trajectories of physical function of all subjects at all three time points (*t*_0_, *t*_1_, and *t*_2_) in the new 2-dimensional coordinate system derived from the PCA. The first follow-up (*t*_0_–*t*_1_) is always presented via a black arrow, the second follow-up (*t*_1_–*t*_2_) in different colors in dependence of its cluster membership (please see the legend).

Table 4 shows the characteristics of the subjects from the five clusters. Cluster 4 is characterized by the highest age of the subjects (79.2 years) and a high percentage of women (87%). Regarding physical function all tests showed the lowest level in comparison to the other clusters.

**Table 4:** Baseline (*t*_0_) characteristics according to cluster membership [mean (SD)]

Cluster	1	2	3	4	5
*n*	47	33	52	23	65
Age (years)	75.4 (3.7)	73.6 (2.9)	75.1 (3.2)	79.2 (5.1)	74.7 (3.1)
Female %	94	0	9	87	92
HGS (kg)	19.5 (3.8)	44.7 (4.9)	35.8 (4.2)	19.5 (4.4)	25.3 (3.2)
TUG (s)	8.0 (1.3)	8.1 (1.7)	8.6 (1.3)	11.0 (1.5)	7.8 (1.0)
SCPTT (s)	5.6 (0.8)	5.5 (1.2)	5.8 (0.8)	7.4 (1.1)	5.4 (0.7)
SCPTP (W)	200.3 (34.7)	264.0 (60.0)	237.7 (46.4)	169.2 (30.7)	214.3 (37.1)
4mGS (s)	2.7 (0.4)	2.6 (0.5)	2.8 (0.4)	3.4 (0.4)	2.6 (0.3)
5TCR (s)	11.7 (2.1)	12.5 (3.0)	12.8 (3.1)	14.8 (3.9)	11.3 (2.6)
6mWT (m)	441.0 (57.3)	473.4 (74.5)	445.4 (67.3)	343.7 (54.5)	452.3 (55.5)

*HGS* handgrip strength, *SCPTT* stair climb power test (time), *SCPTP* (Power), *TUG* timed up & go, *4mGS* 4 m gait speed, *5TCR* 5 times chair rise, *6mWT* 6 min walk test

**Fig. 2:**
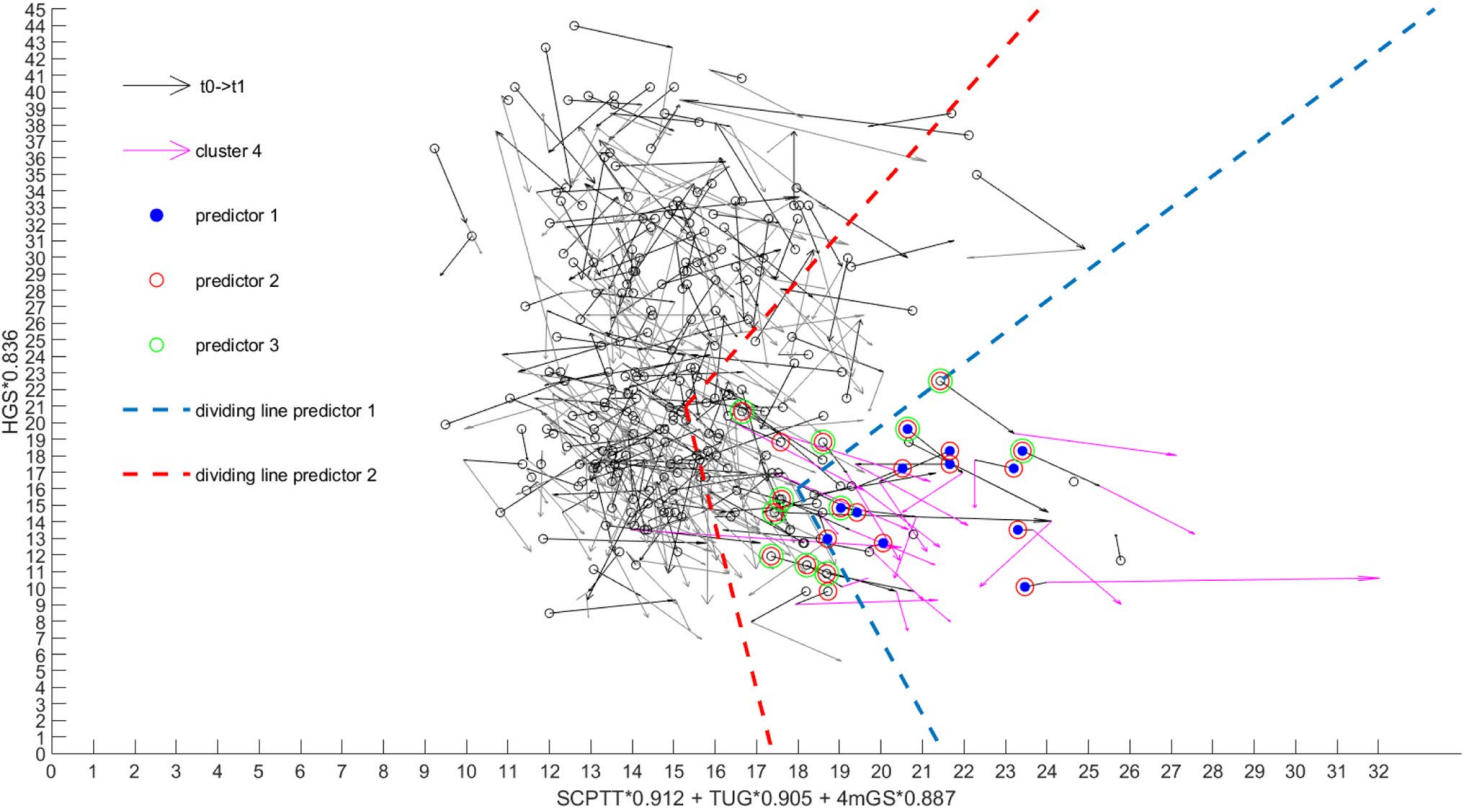
Vector graph showing physical function at all three time points. The relevant cluster is presented (*t*_1_–*t*_2_) in magenta. Dividing lines of predictor 1 and 2 are shown in blue (predictor 1) and red (predictor 2). In accordance to the legend the identified subjects of the three predictors are presented in different colors. As predictor 3 is defined as predictor 2 with the additional condition of negative delta values from *t*_0_ to *t*_1_, no extra dividing lines are able to present. Identified subjects of predictor 2 (*n* = 22) involve those of predictor 1 (*n* = 12); predictor 3 is a subset of predictor 1 and 2 (*n* = 11)

We calculated sensitivity and specificity of the three predictors by testing how many subjects would have been clustered to cluster 4 at baseline (see Fig. 2). With this approach predictive value of the three predictors can be derived as cluster 4 identifies subjects with the lowest physical function. Figure 2 shows the dividing lines of predictor 1 (blue) and 2 (red). Cluster 4 is colored in magenta. In accordance to the legend, subjects who were plotted at baseline and were additionally identified positively with predictor 1, 2 or 3 were colored differently (blue for predictor 1, red for predictor 2, and green for predictor 3). In addition to the figure, Table 5a, b, c present the results of sensitivity and specificity. Predictor 2 showed highest values of sensitivity as 22 subjects of the 23 subjects of cluster 4 were also identified at baseline. Predictor 1 missed 11 and predictor 3 missed 12 subjects and exhibit therefore a sensitivity of 52%, respectively 48%. Figure 2 shows the dividing lines of predictors 1 and 2 (predictor 3 was defined as a negative delta value of the vector positions, meaning deterioration of physical function, from *t*_0_–*t*_1_ plus dividing lines of predictor 2, therefore extra dividing lines are not available). To judge assessment results in clinical settings regarding the risk of a person* i* to be a low-performer (“cluster 4”), the following coordinates [*t*_0_^*x*^(*i*), *t*_0_^*y*^(*i*)] have to be calculated:

**Table 5:** Predictor 1, Predictor 2, Predictor 3

	Cluster 4	Other clusters	Total
Predictor 1^a^			
Test positive	12	3	15
Test negative	11	194	205
Total	23	197	220
Predictor 2^b^			
Test positive	22	36	58
Test negative	1	161	162
Total	23	197	220
Predictor 3^c^			
Test positive	11	3	14
Test negative	12	194	206
Total	23	197	220

^a^Sensitivity = 12/23 = 52%, specificity = 194/197 = 98%

^b^Sensitivity = 22/23 = 96%, specificity = 161/197 = 82%

^c^Sensitivity = 11/23 = 48%, specificity = 194/197 = 98%

*t*_0_^*x*^(*i*) = SCPTT*0.912 + TUG*0.905 + 4mGS*0.887 and *t*_0_^*y*^(*i*) = HGS*0.836.

Based on this individual coordinate of a person, the following conditions have to be checked in order to decide on cluster 4 membership:

*x*_min_ = [(*t*_0_^*y*^(*i*) − 97.282)/− 4.52] < *t*_0_^*x*^(*i*) and *y*_min_ = 1.887*t_0_^*x*^(*i*) − 17.931 > *t*_0_^*y*^(*i*).

## Discussion

The purpose of this research was to describe the longitudinal physical performance of older community-dwelling adults above the age of 70 over 2 years, and to develop a data-driven model to reduce comprehensive geriatric assessment to the most relevant tests that predict individual physical decline of subjects of the observational study VERSA within the primary prevention project AEQUIPA. To this end, inclusion criteria were initially selected with the idea of the subjects being able to participate in intervention action beyond domesticity; we therefore addressed the fittest older adults in our area. The cohort of 251 community-dwelling people (mean age 75.4 years) initially exhibited physical limitations only occasionally. Within the observational period of two years, the majority of the cohort were physically stable in terms of strength, power, balance and endurance, starting from a high level of performance for this age group. Considering the mean values of the present assessment, handgrip in women after two years was close to the BMI-associated cut-offs for low HGS [22]. In 5TCR, the subjects did not score the maximal 4 points in SPPB requiring a duration of under 11.7 s with a mean of 12.4 s initially and 11.1 s after 2 years. The variables with the highest percentage changes after 6 months were 5TCR and 6mWT; those after 24 months were HGS and SCPTT. The divergent result of the development of 5TCR over 2 years has to be discussed. Generally, the regular assessment of physical performance should be considered as low-grade intervention because it motivates the largely fit cohort of older people to perform well in the tests. We assume that the subjects started to exercise before the study commenced, as we experienced a highly motivated and interested study group. However, the effect was not sustained throughout the study period and was only observed for 5TCR by the end of the study.

Many tests of the geriatric assessment exhibit ceiling effects in high-performers. Nevertheless, the developed method enabled us to identify subjects with lowest function. The subjects with the lowest values of physical performance were identified by clustering (Cluster 4) based on a reduced comprehensive geriatric assessment containing the most relevant mobility and strength tests (SCPTT, TUG, 4mGS and HGS). These subjects were close to the threshold for relevant functional decline, or exceeded it; the majority were women (87%). They exhibited a greater decline of HGS (*Y*-axis) than men, and started out, as expected, from a lower level. We developed a predictor that identifies, with a very high degree of sensitivity (96%) and specificity (82%), subjects who were grouped in Cluster 4. This predictor considered the baseline values as we used the means values of the vectors under consideration of its cluster membership at *t*_2_ to predict the assignment to cluster 4 (on the basis of follow-up data). Regarding clinical relevance, patient’s data on HGS, SCPTT, TUG and 4mGS can be judged by using the presented equation which describes the conditions to be localized within the dividing lines of the predictor, meaning that all measurements in clinical settings can be assessed with regard to the risk of low physical function. “Cluster 4 subjects” show the highest deterioration and lowest baseline levels in terms of function. Since the data were collected within a primary prevention project, no endpoints such as “hospitalization”, “death” or “sustainable disability” were relevant, and they occurred only rarely. Identifying older adults in need of prevention at an early stage is most important in this context. After all, identifying a group for which participating in a fitness program (e.g. FITT) could prevent deterioration was the downstream key target. Further intervention studies are required to determine whether intervening at an early stage in the identified group by encouraging them to participate in fitness programs would prolong their independence and increase their quality of life. We believe that by choosing specific tools from the established geriatric assessment, which was developed for use in an extremely heterogeneous population, a high predictive quality can also be achieved for a functionally high-performing group of older adults. Also technology-based approaches provide high potential in this regard [28]. Identification of persons at risk in this apparently competent group would enable primary prevention interventions to postpone functional disability and need of care.
